# Depression and quality of sleep in patients with type 1 diabetes being under regular diabetes care

**DOI:** 10.1192/j.eurpsy.2023.757

**Published:** 2023-07-19

**Authors:** K. Cyranka, B. Matejko, A. Chrobak, D. Dudek, B. Kieć- Wilk, K. Cyganek, P. Witek, M. Małecki, T. Klupa

**Affiliations:** 1 Jagiellonian University Medical College, Cracow; 2 Jagiellonian University Medical College; 3 Jagiellonian University Medical College, Krakow, Poland

## Abstract

**Introduction:**

Research indicates that co-morbid diabetes and depression is common; however, the implications for clinical practice remain unclear

**Objectives:**

The aim of the study was to check the prevalence of depression in patients with T1DM who are provided with optimal conditions of diabetes care and to identify possible risk factors connected with affective traits

**Methods:**

Out of the 107 patients, 78 (54 females, 24 males) were included for the analysis (HbA1c [%] 7.11±1.0, BMI [kg/m2] 25.3, ± 5.6; Years of T1DM [N] 13.7±8.3). The patients filled in a set of questionnaires during their regular visit to the outpatient clinic. Three patients from the whole group were on intensive insulin therapy with Multiple Daily Injections (MDI) and Self-Monitoring of Blood Glucose (SMBG), all the rest were on various types of personal insulin pumps (years on insulin pump [N] 9.1±4.5). All the patients were on regular diabetologist care, with regular visits in a Centre for Advanced Technologies in Diabetes (at least every 6 months).

**Results:**

In QIDS-S 26 patients (33.8%) were screened positive for depression, in PHQ 57.7% of the patients (45 patients) had symptoms of depression (age was negatively correlated with PHQ score (r= -0.26; p=0.023)). In CES-D 16 (20%) of the patients assessed their present affect as depressed. Quality of sleep was highly correlated with depressive symptoms CESD (r=0.61, p=0.001), PHQ Score (r=0.62; p=0.001), QISD (r=0.68; p=0.001).

**Conclusions:**

The prevalence of affective disorders and poor sleep quality in the examined T1DM patients was much higher than in the general population. Even if the patients have in general good glycemic control, their mental health condition should not be neglected. Well organized cooperation between patients, diabetologists, psychiatrists and psychotherapists is needed.

**Disclosure of Interest:**

None Declared

